# New Insights into Properties of Methanol Transport in Sulfonated Polysulfone Composite Membranes for Direct Methanol Fuel Cells

**DOI:** 10.3390/polym13091386

**Published:** 2021-04-24

**Authors:** Cataldo Simari, Isabella Nicotera, Antonino Salvatore Aricò, Vincenzo Baglio, Francesco Lufrano

**Affiliations:** 1 Department of Chemistry and Chemical Technologies, University of Calabria, 87036 Arcavacata di Rende (CS), Italy; isabella.nicotera@unical.it; 2 CNR-ITAE, Istituto di Tecnologie Avanzate per l’Energia “Nicola Giordano”, Via Salita S. Lucia sopra Contesse n., 5-98126 S. Lucia-Messina, Italy; antonino.arico@itae.cnr.it (A.S.A.); vincenzo.baglio@itae.cnr.it (V.B.)

**Keywords:** direct methanol fuel cells, PFG-NMR, sulfonated polysulfone, methanol crossover, acidic silica

## Abstract

Methanol crossover through a polymer electrolyte membrane has numerous negative effects on direct methanol fuel cells (DMFCs) because it decreases the cell voltage due to a mixed potential (occurrence of both oxygen reduction and methanol oxidation reactions) at the cathode, lowers the overall fuel utilization and contributes to long-term membrane degradation. In this work, an investigation of methanol transport properties of composite membranes based on sulfonated polysulfone (sPSf) and modified silica filler is carried out using the PFG-NMR technique, mainly focusing on high methanol concentration (i.e., 5 M). The influence of methanol crossover on the performance of DMFCs equipped with low-cost sPSf-based membranes operating with 5 M methanol solution at the anode is studied, with particular emphasis on the composite membrane approach. Using a surface-modified-silica filler into composite membranes based on sPSf allows reducing methanol cross-over of 50% compared with the pristine membrane, making it a good candidate to be used as polymer electrolyte for high energy DMFCs.

## 1. Introduction

Direct methanol fuel cells (DMFCs) are envisaged as powerful systems for next generation electronic devices, capable to sustain longer operation compared to Li-batteries without the drawbacks of the time-consuming charging process [1,2,3,4,5]. DMFCs utilize a polymer electrolyte membrane (PEM) as the electrolyte and separator between anode and cathode; the proton conductivity and methanol permeability of this latter are among the key factors limiting the DMFC performance, whereas the membrane cost and durability greatly influence the potential commercialization of complete devices [6,7,8]. State-of-the-art membranes for DMFCs are based on perfluorosulfonic acid membranes (PFSAs), such as Nafion^®^ membranes, which are used successfully in DMFCs operating with a low methanol concentration (1 or 2 M) at the anode [9,10]. Operation with high methanol concentration produces high methanol permeation through the membrane, from the anode to the cathode, leading to a loss of fuel efficiency in the DMFC together with a mixed potential at the cathode with a consequent decrease of cell voltage, unless a cathodic catalyst tolerant to the alcohol is used [9,11,12]. Consequently, the research on new proton exchange membranes is mandatory not only to reduce the methanol permeation while maintaining a good proton conductivity, but also to reduce the cost compared to the expensive PFSA membranes.

A series of different strategies are currently pursued to find polymer electrolytes able to replace PFSA membranes, maintaining or improving the performance of the DMFC, also at higher methanol concentrations to achieve higher energy densities. These approaches are based on the use of sulfonated aromatic polymers (SAPs) and their modifications [13,14,15,16,17]. The SAP membranes may exhibit high proton conductivity, high fuel cell performance and suitable durability compared to PFSA membranes. Furthermore, the development of inorganic–organic composite membranes based on SAPs for application in DMFCs can (i) improve the self-humidification of the membrane by dispersing hydrophilic inorganic additives in the polymer [18,19,20,21,22]; (ii) reduce the fuel (methanol) crossover through the membrane [23,24,25,26,27,28]; and (iii) improve the mechanical strength of the membranes without compromising proton conductivity [29,30]. 

The SAPs could be prepared with a high degree of sulfonation, which is highly desirable to achieve a high conductivity. However, this can be accompanied by undesirable higher swelling (or even solubility in hot water) of the membrane and loss of mechanical strength. The addition of an inorganic component into polymer electrolytes is envisaged to compensate these effects, improving the mechanical and chemical stability features and enhancing the thermal stability, proton conductivity and, likely, electrochemical performance [31,32,33,34,35,36]. A further strategy is the use of composite sulfonated aromatic membranes with low degree of sulfonation and low water/methanol swelling, modifying and optimizing the characteristics of the inorganic fillers, i.e., sulfonation, acid groups, etc. [37,38]. This approach was successfully adopted, as demonstrated in previous papers [37,38], in which an investigation of bare and acidic silica as fillers for sulfonated polysulfone membrane was carried out. The membrane properties were adjusted according to the added fillers; in particular, the acidic-silica-based composite membrane exhibited the best electrochemical performance compared to that with the untreated silica and unmodified sulfonated polysulfone membranes. Moreover, the study demonstrated that this developed sulfonated aromatic polymer can be adapted, in such a way, to exhibits low water uptake, low methanol swelling, reduced methanol crossover, high proton conductivity and suitable DMFC performance. Instead, the aim of this work is to investigate the water and methanol transport characteristics of these membranes (both pristine and composites), particularly when a high concentration of methanol (5 M) is used. For this scope, ^1^H-PFG NMR technique was used in this study. Furthermore, an analysis of methanol cross-over and direct methanol fuel cell performance was performed using a high methanol concentration (5 M), in order to validate the used approach under conditions closer to practical DMFC applications.

## 2. Experimental

The acidic silica material was prepared starting from CAB-O-SIL EH-5 silica (Cabot Corporation, Boston, MA, USA), according to a procedure reported in detail elsewhere [37,38,39]. Briefly, silica (20.0 g) was reacted with 4.5 mL of chlorosulfonic acid (concentrate) under stirring over a period of 30 min at room temperature. After complete release of HCl (gas), the obtained white solid material was dried at room temperature and stored in a desiccator. 

A commercial polysulfone polymer (Lati SpA, Varese, Italy) was sulfonated in chloroform (Sigma-Aldrich, Milano, Italy) solution (8 wt./v.%) at 50 °C per 6 h using trimethylsilyl chlorosulfonate (Sigma-Aldrich) as the sulfonating agent and under reflux to produce a silyl sulfonate polysulfone. Thereafter, it was treated with sodium methoxide/methanol solution (30 wt%) at 50 °C for 1 h to obtain a sodium sulfonated polysulfone [40]. At the end, a white fine precipitate was recovered and dried at 70 °C, for 24 h under vacuum. 

The bare membrane was prepared by casting method from sulfonated polysulfone solution in dimethylacetamide (15 wt%); the polymer solution was spread on a glass plate using a manual stainless-steel film applicator. The cast membrane was allowed to evaporate for the solvent removal at 50 °C for at least 15 h. Composite membranes were prepared in the same way by adding 10 wt% acidic or bare silica to the polymeric dispersion. Membranes of uniform thickness (100 m) were prepared. Ion-exchange capacities (IECs) of the membranes were determined by back titration; the values are 1.37, 1.32 and 1.34 mmol/g for the bare sulfonated polysulfone (SPSf), composite silica-SPSf (SPSf-SiO_2_) and composite acidic silica-SPSf (SPSf-SiO_2__sulf), respectively. 

**Figure 1:**
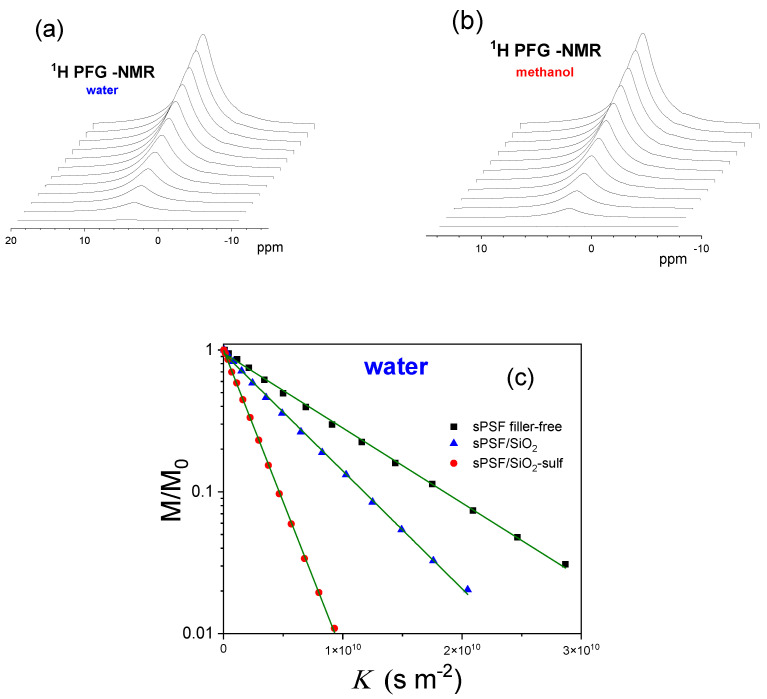
^1^H PFG NMR spectra corresponding to (**a**) water and (**b**) methanol absorbed in the Pebax membrane at 20 °C. (**c**) Plot of the normalized peak areas vs. *K* ((γgδ)^2^ (Δ−δ/3)) for water confined in the three membranes. The solid green line represents the linear fitting to the experimental data.

NMR measurements were performed with a Bruker NMR spectrometer AVANCE 300 Wide Bore working at 300 MHz on ^1^H (Bruker, Milan, Italy), equipped with multinuclear Diff30 Z-diffusion probe for pulse field gradient (PFG) measurements. Self-diffusion coefficients of water and methanol were performed by using the pulsed field gradient spin−echo (PFGSE) method [41]: the sequence consists of three 90° rf pulses (π/2-τ_1_-π/2-τ_m_-π/2) and two gradient pulses that are applied after the first and the third rf pulses, respectively. The echo is found at time τ = 2τ_1_ + τ_m_. For illustrative purposes, the ^1^H PFG NMR spectra corresponding to water and methanol absorbed in sPSf filler-free are shown as a function of the amplitude of the field gradient in Figure 1a,b, respectively. Following the usual notation, the magnetic field gradient pulses have magnitude *g*, duration *δ*, and time delay Δ. The FT echo decays were analyzed by means of the relevant Stejskal–Tanner expression:

(1) $$I=I_{0}e^{-KD}$$

where, *I* and *I*_0_ represent the intensity/area of a selected resonance peak in the presence and in absence of gradients, respectively. *K* is the field gradient parameter, defined as:

(2) $$K=\;{\left(\gamma g\delta \right)}^{2}\left(\Delta -\;\frac{\delta }{3}\right)]$$

In these experiments, *δ* and Δ were kept at 0.8 ms and 8 ms, respectively, the echo time was 5.7 ms, while *g* was varied between 100 and 800 G cm^−1^, incremented in 12 steps. The number of scans for the ^1^H PFG-NMR measurements were 4, while the number of repetitions was kept equal to 2. The repetition time was about 5 times the longitudinal relaxation time (*T*_1_) and the total acquisition time ranged between 2 and 10 min. Figure 1c illustrates the experimental echo-signal attenuation curves in the diffusion measurements for water and methanol in the three membranes. On the basis of the very high SNR (signal to noise ratio), the attenuation higher than 90%, the low standard deviation of the fitting curve and repeatability of the measurements, the uncertainties in the PFG-NMR self-diffusion measurements were calculated to be ca. 3%. Measurements were collected as a function of methanol concentration (e.g., 1–5 M CH_3_OH) and temperature [42].

The electrodes used in this investigation were prepared as previous described [37,38]. A catalytic ink, consisting of an unsupported Pt-Ru (1:1 atomic ratio, Johnson-Matthey), with a Pt loading of 2.5 mg cm^−2^, and a 15 wt% Nafion ionomer (Ion Power, 5 wt% solution), was employed at the anode, while a Pt black (Johnson-Matthey, London, UK), with the same amount of metal and Nafion ionomer as the anode, was used at the cathode. The membrane-electrode assemblies (MEAs) were obtained by hot-pressing the electrodes onto the membrane at 90 °C and 30 kg·cm^−2^ for 5 min. The MEAs were tested in a 5 cm^2^ single cell using an Autolab PGSTAT 302 Potentiostat/Galvanostat (Metrohm Italia, Origgio (MI), Italy) equipped with an FRA module of impedance. The methanol solution was fed at the anode with a flow rate of 3 mL min^−1^, whereas dry air was fed at the cathode (100 mL min^−1^). Area specific resistance and ionic conductivity of the investigated polysulfone membranes were obtained by electrochemical impedance spectroscopy (EIS). The EIS measurements were performed in a frequency range from 100 kHz to 0.1 Hz on the cells kept at 0.3 V. Under these conditions, the fuel cell operates in the ohmic region. The amplitude of the sinusoidal excitation signal was 10 mV. The series resistance was determined from the high frequency intercept on the real axis in the Nyquist plot.

The crossover measurements, using a 5 M methanol solution, were carried out by linear sweep voltammetry (LSV) experiments at a scan rate of 2 mV·s^−1^ and in the voltage range from 0 to 0.95 V. A 5 M MeOH solution (3 mL·min^−1^) was fed to one side of the cell, used as the counter/reference electrode and He (100 cm^3^·min^−1^) was supplied to the other compartment (working electrode). Methanol crossing the membrane is oxidized at the working electrode generating a positive current, which reaches a plateau when all methanol is converted to CO_2_ under steady state conditions [43,44,45].

## 3. Results and Discussions

**Figure 2:**
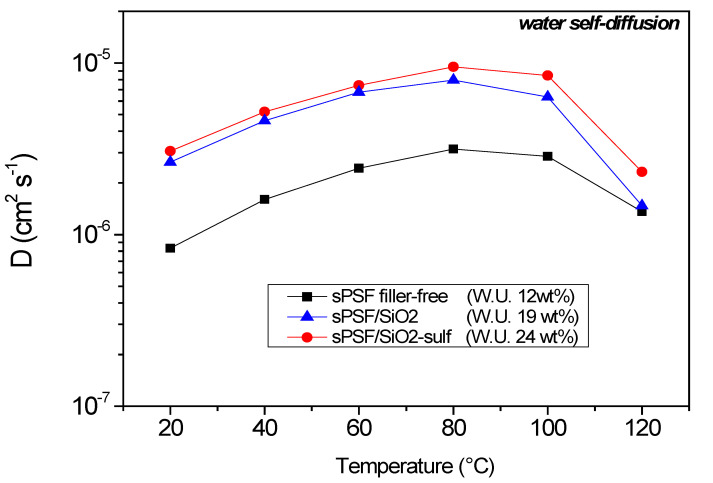
Water self-diffusion coefficients as a function of temperature for the different polysulfone membranes.

In this study the ^1^H-PFG NMR technique was used to investigate the transport properties of water and methanol molecules confined in the porous structure of sPSf and composite membranes, and to check the effect of the inorganic fillers added. Firstly, water self-diffusion coefficients were measured on completely hydrated membranes, and the data are displayed in Figure 2 for the temperature range from 20 to 120 °C. As observed, the amount of water absorbed from the three membranes was quite different, and already from these data emerged the hydrophilic effect of the silica nanoparticles dispersed: the sPSf pristine membrane shows a water uptake of about 12 wt.%, while in the composites it significantly increased up to about 19 wt.% for the sPSf-SiO_2_ composite and up to 24 wt% for the sPSf-SiO_2__sulf. Evidently, the different water content affected the diffusion coefficients, especially in the region of low temperatures, i.e., below 80 °C, where the water evaporation is negligible. Indeed, both composites promote higher mobility of the water molecules with coefficients 3 times higher than the filler-free membrane. In all cases, the self-diffusion, *D*, increased as the temperature increased due to the thermal motions, but at 80 °C the thermal energy was no more able to compensate the effect caused by the water evaporation: diffusion started to fall because most of the “free water” was lost from the membranes, and remained the hydration water to the hydrophilic groups, whose diffusivity was clearly inhibited. 

The analysis of the temperature dependence of the water diffusivity, using Arrhenius equation in the temperature range in which the diffusion increased, i.e., 20–80 °C, gives the corresponding activation energy (*E_a_*) of the diffusion process, which is the energy barrier for carrier transfer from one free site to another one. Both the composites’ membranes have E_a_ values very similar, 3.82 kcal/mol for sPFS-SiO_2_ and 3.87 kcal/mol for sPFS-SiO_2__sulf, which are typical values usually obtained also for Nafion-based electrolytes [46,47]. Instead, the sPSF pristine membrane shows a much higher value, 4.6 kcal/mol, likely associated with a low hydration level inside the hydrophilic pores of this membrane: the water molecules are strongly interacting with the sulfonic acid groups and the structural diffusion (Grotthuss proton transport mechanism) is the preferential path for proton diffusivity respect to the vehicular mechanism. The hopping mechanism foresees the reorientation of the protons in the solid-like sites through the breaking of such electrostatic interaction bonds, therefore the activation energy associated with this motion increases. Concerning the behavior of such membranes with respect to methanol, they were swelled by immersion in aqueous methanol solutions at two different alcoholic concentration, 1 and 5 M. Table 1 shows the uptake values obtained at 20 °C. Compared with the water uptake values seen previously, there are some differences, in particular at higher methanol concentration, reaching for the composites an uptake of about 30 wt%, and 20 wt% for the filler-free sPSf. However, if we compare these values with those usually obtained in Nafion based electrolytes (34 wt% with 4 M methanol) [48], the swelling here is quite lower, confirming a lower affinity of the methanol to polysulfone polymer compared to perfluorosulfonic acid membranes. This parameter is important to overcome the major limitation of using high concentrations of methanol solutions in DMFCs.

To perform the NMR measurements, it was necessary to discriminate between the NMR signals of methanol and water, which, in the case of solvents confined in membranes, due to the linewidth of the ^1^H-NMR signals, is not possible to distinguish, through their chemical shift [48]. Therefore, the membranes were equilibrated in solutions prepared with deuterated molecules, i.e., mixture of CH_3_OD/D_2_O and CD_3_OD/H_2_O. The reason to use CH_3_OD instead of CH_3_OH is due to the fast rate exchange of hydroxyl groups between water and methanol molecules during the “NMR times”, which could affect the measurements. Hence, we used the only signal coming from the methyl groups to perform the NMR diffusometry measurements of methanol confined inside the electrolyte films swelled with CH_3_OD/D_2_O solutions, and water self-diffusion measurements on membranes swelled with CD_3_OD-H_2_O solutions.

**Figure 3:**
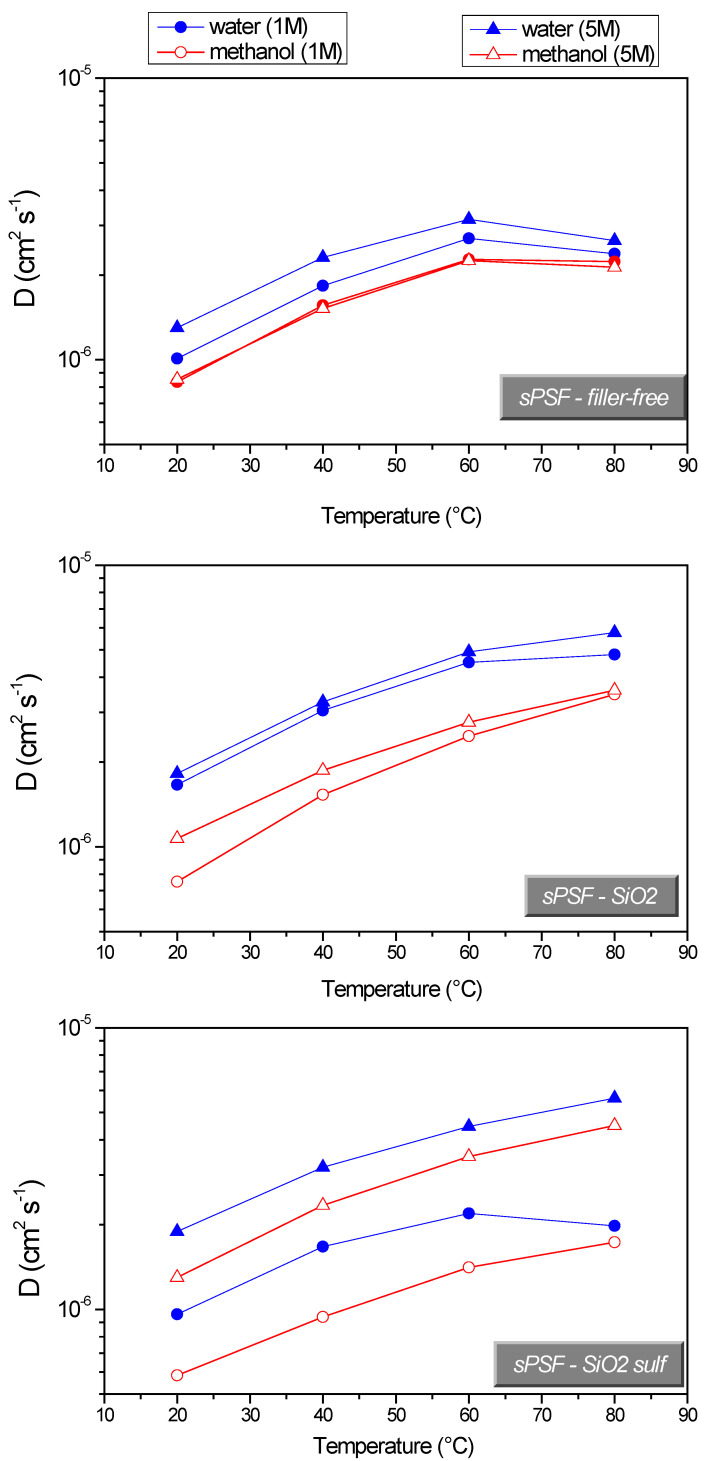
Self-diffusion coefficients of water and methanol in 1 and 5 M solutions confined in filler-free sPSf and the two composites, from 20 up to 80 °C.

Figure 3 shows a comparison of the diffusion coefficients of water and methanol (1 and 5 M solutions) measured in swollen membranes, in the range of temperature 20–80 °C. The most important consideration concerns the higher water diffusion respect to methanol diffusion for all three membranes and for both two solution concentrations. Furthermore, the difference becomes larger for the composites, proving the beneficial methanol blocking effect of the nanoparticles dispersed in the polymer.

**Figure 4:**
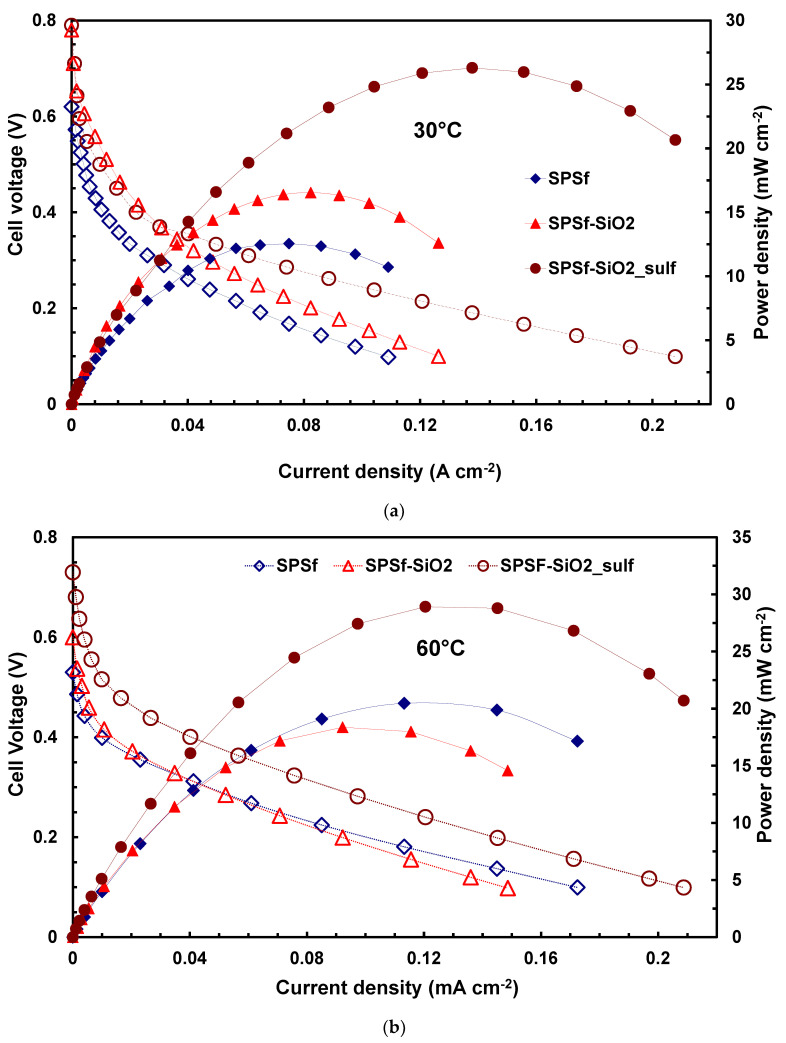
Polarization and power density curves of different polysulfone membranes at (**a**) 30 °C and (**b**) 60 °C and 5 M MeOH.

These good properties regarding the blocking effect of methanol molecules envisage a suitable behavior of these membranes in DMFCs operation with high methanol concentration (5 M). Accordingly, MEAs prepared with the composite and filler-free membranes were investigated in DMFC using 5 M as the anode feed. Figure 4 shows a comparison of the polarization curves for these MEAs at 30 °C (Figure 4a) and 60 °C (Figure 4b), feeding dry air at the cathode side under atmospheric pressure. 

At 30 °C, the best performance, in terms of both power density, current density and open circuit voltage (OCV), was obtained with the MEA based on sPSf-SiO_2__sulf followed by sPSf-SiO_2_ and filler-free SPSf membranes. The OCV for the acidic composite membrane (sPSf-SiO_2__sulf) was 0.79 V, slightly better than that recorded for the sPSf-SiO_2_ (0.77 V) and significantly higher than the value of 0.62 V obtained with the filler-free membrane. A higher OCV is a clear indication of a lower amount of MeOH crossing the membrane and reaching the cathode, since the effect of the mixed potential (oxygen reduction and methanol oxidation) at the latter electrode is limited. The same behavior is also confirmed at higher temperatures (60 °C), although a slight decrease of the OCV was observed for all MEAs. In fact, as known from the literature [23,28], methanol cross-over increases with the temperature, producing a decrease of OCV and performance, although this latter is usually compensated by the enhanced kinetics of methanol oxidation and oxygen reduction reactions. Furthermore, also the proton conductivity of the membrane increases with the temperature leading to an increase of fuel cell performance. Unfortunately, in this study the performance enhancement is not so significant due to the high concentration of methanol (5 M), which produces a large methanol cross-over not counteracted by the presence of pure oxygen at the cathode side (the tests were carried out under more reliable conditions, using dry air at atmospheric pressure at the cathode); thus, methanol competes with oxygen creating a mixed potential, which reduces the fuel cell performance. As a result, the maximum power density achieved with the sPSf-SiO_2__sulf was 26 mW·cm^−2^ at 30 °C, with an increase to 29 mW·cm^−2^ at 60 °C, probably due to the negative effect of methanol crossing the membrane. However, the performance observed with the composite acid filler-based membrane (sPSf-SiO_2__sulf) was significantly higher than that achieved with the other two membranes (Figure 3).

**Figure 5:**
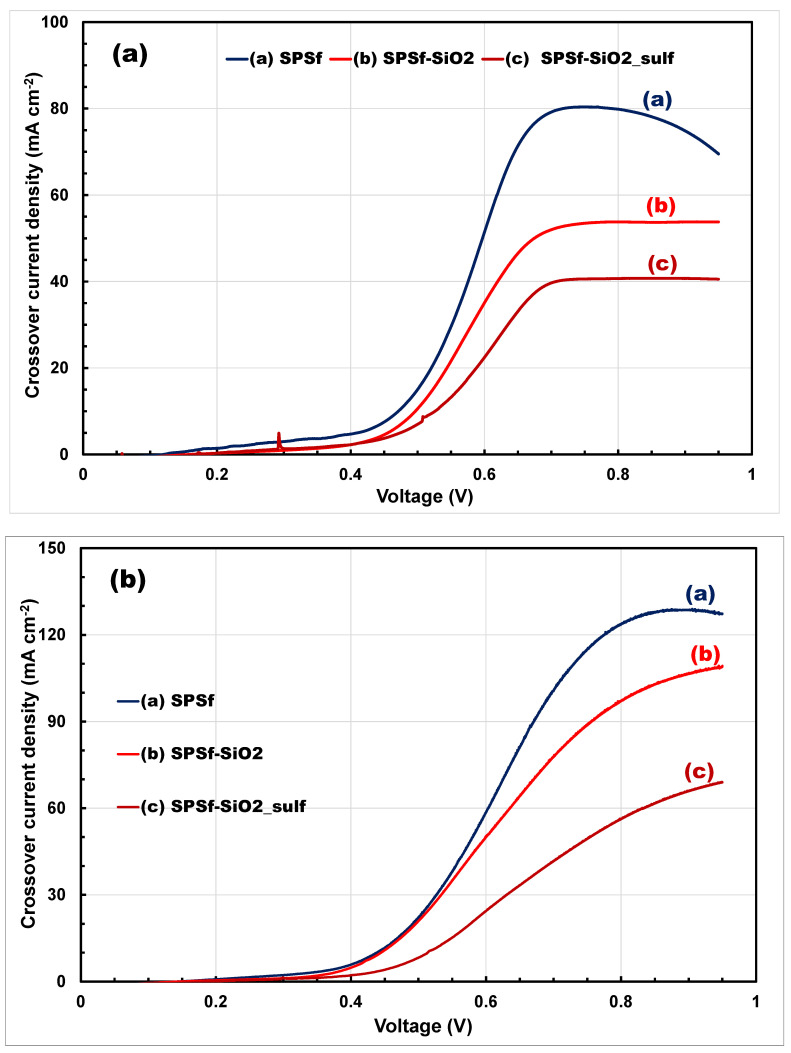
Comparison of methanol crossover current densities, using a 5 M methanol solution, for the SPSf and composite SPSf membranes at (**a**) 30 °C and (**b**) 60 °C.

To confirm the beneficial effect of the composite membranes in reducing methanol permeation through the membrane, methanol crossover measurements, using linear sweep voltammetry, were carried out, both at 30 and 60 °C. The curves are reported in Figure 5.

The methanol cross-over equivalent current (the current resulting from the oxidation of crossed methanol) for the different MEAs confirms the trend of the OCV values, since the lowest current was observed for the sPSf-SiO_2__sulf membrane, with values of 40 and 65 mA·cm^−2^ at 0.9 V and 30 and 60 °C, respectively. The significant differences between this membrane and the other two samples used for comparison could be ascribed not only to the increased diffusion path length of MeOH/H_2_O generally observed in the composite membranes, known as the tortuosity factor, but also to the occurrence of tightly bonded water molecules on the hydrophilic acidic silica (SPSf-SiO_2__sulf) clusters. These results are in line with non perfluorinated based membranes reported in the literature and largely lower than current state-of-the-art Nafion membrane of similar thickness (Nafion 115, 125 µm), which shows a crossover current density of 195 mA·cm^−2^ at 60 °C, feeding the DMFC with a 2 M methanol solution [28,49].

However, the achievement of high DMFC performance depends on many properties of the membrane; not only water and MeOH transport, methanol concentration and permeability of the membrane are important parameters determining the fuel cell behavior. High proton conductivity is an essential indicator for assessing the suitability of a membrane for fuel cell applications.

**Figure 6:**
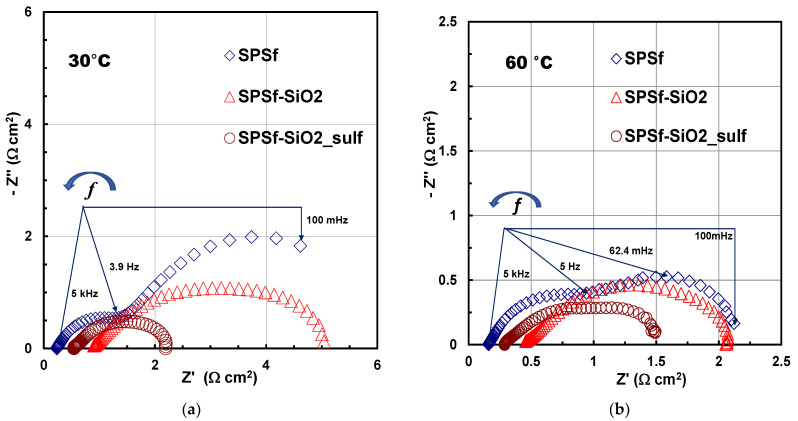
Electrochemical impedance spectra (Nyquist plots) for the MEAs equipped with the different membranes recorded at 0.3 V and 5 M MeOH solution at (**a**) 30 °C and (**b**) 60 °C.

As further study to highlight the different features of the membranes, in-situ electrochemical impedance spectroscopy (EIS) measurements were carried for all MEAs. Figure 6a shows Nyquist plots for the single cells tested under potentiostatic condition at 0.3 V and 30 °C. The total impedance spectra profiles result from the overlapping of two distorted semicircles. The high frequency semicircle occurs in the frequency range from 5 kHz to 4–5 Hz, whereas the low frequency semicircle occurs from 4–5 Hz to 100 mHz. The series resistance (R_s_) values obtained from the high frequency intercept on the *x*-axis were 0.25, 0.54 and 0.90 cm^2^ for the MEAs based on filler-free SPSf, sPSf-SiO_2__sulf and SPSf-SiO_2_, respectively. This indicates that, although the best performance, in terms of power output, in a DMFC is obtained with the composite membrane based on acidic silica followed by that one with bare silica, the presence of an inorganic filler, such as silica, not presenting additional ionic conduction characteristics, decreases the proton conductivity of the SPSf-SiO_2_ membrane. This latter is increased by functionalizing silica with sulfonic acid groups, but not enough to achieve the conductivity of pristine sPSf membrane (Figure 6a).

It is important to point out that the polarization resistance (R_p_) was lower for the sPSf-SiO_2__sulf membrane; this is a further confirmation of the methanol cross-over reduction using this membrane, especially at 30 °C (Figure 6a), but also at 60 °C (Figure 6b). In fact, the contribution of the anode and cathode to the impedance was clearly visible from the two semicircles shown in Figure 6a, being the first one at high frequency attributed more to the anode and second one at low frequency (i.e., 5 Hz to 100 mHz) to the cathode side [50,51,52]. As reported in Reference [53], the amplitude (i.e., diameter) of the low frequency semicircle increases with the methanol concentration due to an increasing methanol crossover. Similarly, in the present case, a large low-frequency semicircle indicates that, beside the slow reaction kinetic of oxygen reduction reaction, there is a strong poisoning due to methanol crossover. Here, the MEA based on SPSf-SiO_2__sulf shows a smaller radius for the low frequency semicircle, which is translated into a lower total R_p_. This indicates that this membrane displays a lower methanol crossover than the other two membranes; accordingly, there is a lower methanol content at the cathode, which could compete with the oxygen reduction reaction.

The same trend related to the charge transfer resistance of membranes is clearly observed by Nyquist plots reported in Figure 6b (at 60 °C), where each radius is the sum of two overlapped distorted semicircles of smaller amplitude, indicating an enhanced kinetic effect of the temperature on the reactions occurring at the cathode and anode. Additionally, the values of cell resistance, obtained at high frequency intercept on the *x*-axis, are lower at 60 °C compared with 30 °C (0.16, 0.30 and 0.48 cm^2^ for the MEAs based on filler-free SPSf, sPSf-SiO_2__sulf and SPSf-SiO_2_, respectively).

**Figure 7:**
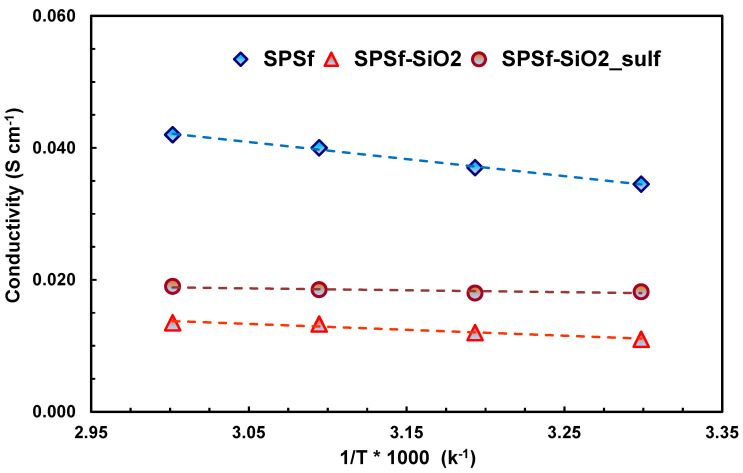
In-situ proton conductivity (T = 30–60 °C) of the different polysulfone membranes in 5 M MeOH solutions.

As above reported, the conductivity of the pristine sPSf was higher than the composite membranes (Figure 7), due to the fact that the introduction of an inorganic filler, showing very low or no proton conductivity at low temperature (30–60 °C), led to a tortuosity increase (the same phenomenon occurring for methanol permeation, but in the case of protons it reduced the conductivity), causing an increase of cell resistance (see Figure 6). The approach of modifying the surface characteristics of silica by sulfonation was demonstrated very useful to increase the proton conductivity, allowing in the future to tailor the sulfonation features of the polymer to design membranes with low methanol permeability (reducing the sulfonation degree of the polymer and using acidic functionalities on the fillers). The present results indicate that the composite membrane based on acidic silica (SPSf-SiO_2__sulf) could potentially be used in high energy density DMFC devices due to a good compromise among low methanol crossover, low swelling due to reduced water/MeOH uptake, good conductivity and suitable DMFC performance.

## 4. Conclusions

Here, a useful approach to mitigate methanol crossover and its impact on DMFC performance was presented. The approach did not involve the use of thicker membranes, as conventionally used to decrease methanol crossover, but a proper tailoring of the characteristics of fillers used for the fabrication of composite membranes. Furthermore, a low cost sulfonated polysulfone was employed to reduce the cost of polymer electrolyte membranes and increase the efficiency of DMFC. The composite membrane based on sulfonated polysulfone and acidic silica allows to reduce methanol cross-over of about 50% respect to the pristine membrane, making it a good candidate to be used as composite polymer electrolyte membrane for direct methanol fuel cell applications.

## Figures and Tables

**Table 1:** Aqueous methanol solution uptakes (wt%) of the membranes at 20 °C.

Membranes	Uptake (wt%) 1 M MeOH Solution	Uptake (wt%) 5 M MeOH Solution
sPSf (filler-free)	14	20
sPSf-SiO_2_	24	30
sPSf-SiO_2__sulf	27	30

## Data Availability

Not applicable.
